# Supplementary material for: Using multiple qualitative methods to inform intervention development: Improving functional status measurement for older veterans in primary care settings
Source: PLoS One. 2023 Aug 24;18(8):e0290741. doi: 10.1371/journal.pone.0290741 (PMC10449158; doi:10.1371/journal.pone.0290741)
Supplement: Attachment — Submitted filename: PLoS One response letter 8.4.23 FINAL 2.docx [file pone.0290741.s001.docx]

Rebecca T. Brown, MD, MPH

Assistant Professor of Medicine

3615 Chestnut Street

Philadelphia, PA 19104

Tel: (215) 573-5563

Rebecca.Brown@pennmedicine.upenn.edu

August 4, 2023

Mobolanle Balogun, MBBS, MPH, FMCPH, FWACP
Academic Editor
PLOS ONE

Dear Dr. Balogun,

We appreciate the opportunity to address the Editors’ and Reviewers’ comments and revise our manuscript. Below, please find item-by-item responses to the Reviewers’ comments, which are included verbatim. All page and paragraph numbers refer to locations in the revised manuscript. Attached please find the revised manuscript. We have tracked all changes to indicate the revised portions.

**Responses to Journal Requirements:**

When submitting your revision, we need you to address these additional requirements.

1. Please ensure that your manuscript meets PLOS ONE's style requirements, including those for file naming. The PLOS ONE style templates can be found at <https://journals.plos.org/plosone/s/file?id=wjVg/PLOSOne_formatting_sample_main_body.pdf> and <https://journals.plos.org/plosone/s/file?id=ba62/PLOSOne_formatting_sample_title_authors_affiliations.pdf>
   a. **Response:** We have formatted the manuscript to meet PLOS ONE’s style and naming requirements.
2. In your Data Availability statement, you have not specified where the minimal data set underlying the results described in your manuscript can be found. PLOS defines a study's minimal data set as the underlying data used to reach the conclusions drawn in the manuscript and any additional data required to replicate the reported study findings in their entirety. All PLOS journals require that the minimal data set be made fully available. For more information about our data policy, please see <http://journals.plos.org/plosone/s/data-availability>.

"Upon re-submitting your revised manuscript, please upload your study’s minimal underlying data set as either Supporting Information files or to a stable, public repository and include the relevant URLs, DOIs, or accession numbers within your revised cover letter. For a list of acceptable repositories, please see <http://journals.plos.org/plosone/s/data-availability#loc-recommended-repositories>. Any potentially identifying patient information must be fully anonymized.

Important: If there are ethical or legal restrictions to sharing your data publicly, please

explain these restrictions in detail. Please see our guidelines for more information on what we consider unacceptable restrictions to publicly sharing data: <http://journals.plos.org/plosone/s/data-availability#loc-unacceptable-data-access-restrictions>. Note that it is not acceptable for the authors to be the sole named individuals responsible for ensuring data access.

We will update your Data Availability statement to reflect the information you provide in your cover letter.

a. **Response:** The underlying data for this study consists of in-depth, qualitive interviews with (1) Veterans with aging-related functional impairment and their caregivers and (2) employees of the U.S. Veterans Health Administration. It is not possible to create a minimal data set with this qualitative data as this study did not obtain ethical approval or informed consent from participants to publicly share underlying qualitative data sets. Relevant excerpts from transcripts are included within the paper. The datasets generated and/or analyzed during this study are not publicly available but may be available upon request at the Center for Health Equity Research and Promotion of the U.S. Department of Veterans Affairs administrative offices, at [215-823-5817](mailto:215-823-5817).

3. Please review your reference list to ensure that it is complete and correct. If you have cited papers that have been retracted, please include the rationale for doing so in the manuscript text, or remove these references and replace them with relevant current references. Any changes to the reference list should be mentioned in the rebuttal letter that accompanies your revised manuscript. If you need to cite a retracted article, indicate the article’s retracted status in the References list and also include a citation and full reference for the retraction notice.
   a. **Response:** We have reviewed the reference list to ensure that it is correct and complete. We do not include any retracted articles.

**Responses to Reviewer #1:**

1. Overall, the paper is well written and focuses on developing an intervention for effective assessment of functional status for older veterans. The intervention was the outcome of multiple qualitative methods of different stakeholders. Strengths- This is an important work with a clear focus and the qualitative approach allows for better exploration. Weakness- For a study that was set out to develop interventions, a more rigorous qualitative analysis may be expected beyond a rapid analysis.
   a. **Response:** We thank the reviewers for these comments. We used multiple approaches to qualitative analysis, including both rapid analysis and thematic analysis. Rapid analysis is increasingly utilized in health services research contexts (e.g., Averill, 2002, Hamilton, 2013, Nevedal et al, 2021). Our use of a matrix approach to team-based rapid analysis allowed us to analyze interviews concurrently with data collection to inform development of process maps and quickly obtain stakeholder feedback. Matrix analysis, which has been found to be as effective and rigorous as traditional deductive analysis using the Consolidated Framework for Implementation Research (Nevedal et al, 2021), was an appropriate approach to meet our study objectives. We also used thematic analysis of primary care provider interviews to contextualize barriers and facilitators to routine measurement, including deductive and inductive coding. In addition, we used an inductive approach to qualitative thematic analysis of patient interviews to understand experience with and preferences for measurement. For further description of the use of these multiple qualitative methods, see Abstract, page 5; Methods, page 6, paragraph 1; Fig. 1 Flowchart of Methods for Intervention Development; and Methods, pages 9-10.
2. Two concerns I have are:

   1. The end-users (i.e. the patients and the caregivers) were only recruited from site 7 (Table 1). As critical stakeholders, the selection of end-users from only one site may limit the generalizability of feedback. The authors mentioned that the 7 sites were diverse- it would be helpful for a global readership to understand better why this selection for the end-users or include as a limitation.

   2. Secondly, it would be important to state if the interventions developed were co-designed or not. If they were, more details would be helpful on other stakeholders who contributed, and if not, the implications of this.
   a. **Response:** We thank the Reviewer for these comments. We recruited the patients and caregivers from a single site where the investigators worked in order to allow for home-based interviews and to facilitate participation among patients with functional impairment. Importantly, this site had a routine, standardized process for measuring functional status which enabled patients and caregivers to discuss their experience and provide feedback on functional status measurement in the clinic. We agree that this may limit the generalizability of the findings from this stakeholder group and have added this as a limitation as follows:

Discussion, page 28, paragraph 2: “We recruited patients and caregivers from a single VA medical center where the investigators worked; while this approach allowed for home-based interviewed and facilitated participation among individuals with functional impairment, the findings for patients and caregivers may be less representative than those of participants recruited from 6 sites nationally.”

Regarding whether the intervention was co-designed, we did not use a formal co-design process in which end-users were involved in the study planning phase or helped determine the research agenda. However, we did strive to incorporate end-user feedback throughout the intervention design process. Specifically, qualitative interviews focused on barriers and facilitators to measuring functional status in primary care were used to inform the design of the intervention. Staff at each staff also reviewed the process maps of functional measurement for their site. In addition, we completed usability testing and iterative refinement of the electronic screening tool with intended users (licensed vocational nurses and PCPs). The use of qualitative interviews is described throughout the methods; we describe the usability testing process as follows:

Methods, page 10, paragraph 2: “The VA Office of Human Factors Engineering completed Human Factors analysis of the screening tool. We then conducted usability testing and iterative refinement with intended users (licensed vocational nurses [LVNs] and PCPs).”

**Responses to Reviewer #2:**

1. Reviewer #2: This article effectively utilizes qualitative methods to investigate the implemntation of new measures designed to ensure the full scale of functional status for older veterans is accurately captured and applied in health care settings. Data is well grounded in semi-structured interviews across multiple VA geographical regions. This article effectively utilizes matrix analysis to locate central themes and reveal compelling findings about the screening and assessment of older adults.
   a. **Response:** We thank the reviewer for these comments.

We thank the Editor and Reviewers for their time and efforts to improve our manuscript.

Sincerely,

Rebecca Brown, MD, MPH
